# Endothelial Transcytosis in Acute Lung Injury: Emerging Mechanisms and Therapeutic Approaches

**DOI:** 10.3389/fphys.2022.828093

**Published:** 2022-03-31

**Authors:** Joshua H. Jones, Richard D. Minshall

**Affiliations:** ^1^Department of Pharmacology, University of Illinois College of Medicine at Chicago, Chicago, IL, United States; ^2^Department of Anesthesiology, University of Illinois College of Medicine at Chicago, Chicago, IL, United States

**Keywords:** caveolae (caveolin-1), endocytosis, Src signaling, PV-1, endothelial permeability, acute lung injury

## Abstract

Acute Lung Injury (ALI) is characterized by widespread inflammation which in its severe form, Acute Respiratory Distress Syndrome (ARDS), leads to compromise in respiration causing hypoxemia and death in a substantial number of affected individuals. Loss of endothelial barrier integrity, pneumocyte necrosis, and circulating leukocyte recruitment into the injured lung are recognized mechanisms that contribute to the progression of ALI/ARDS. Additionally, damage to the pulmonary microvasculature by Gram-negative and positive bacteria or viruses (e.g., *Escherichia coli*, SARS-Cov-2) leads to increased protein and fluid permeability and interstitial edema, further impairing lung function. While most of the vascular leakage is attributed to loss of inter-endothelial junctional integrity, studies in animal models suggest that transendothelial transport of protein through caveolar vesicles, known as transcytosis, occurs in the early phase of ALI/ARDS. Here, we discuss the role of transcytosis in healthy and injured endothelium and highlight recent studies that have contributed to our understanding of the process during ALI/ARDS. We also cover potential approaches that utilize caveolar transport to deliver therapeutics to the lungs which may prevent further injury or improve recovery.

## Introduction

Acute Lung Injury (ALI) and its more severe form, Acute Respiratory Distress Syndrome (ARDS) are characterized by hypoxemic respiratory failure of both lungs, preventing tissue oxygenation that may result in multi-organ dysfunction and death (Matthay et al., 2019). ALI/ARDS results from numerous etiologies, including infectious agents, trauma, pancreatitis, and transfusion of blood products. While some affected individuals have a mild course of injury and recover, many require mechanical ventilation and additional treatment within intensive care units. Unfortunately, there is no cure for ARDS and the mainstay of treatment is supportive care (Fan et al., 2018). Prior to the COVID-19 pandemic, the available data estimated that there were roughly 190,000 cases of ARDS in the United States each year (Rubenfeld et al., 2005). In the wake of the COVID-19 pandemic and the subsequent increase in the incidence of ARDS, there is a renewed interest in understanding the underlying mechanisms contributing to the pathogenesis and progression of ARDS, which may lead to novel interventions that improve recovery and survival outcomes (Matthay et al., 2020).

Pneumonia and non-pulmonary sepsis remain the leading causes of ARDS (Pham and Rubenfeld, 2017). Sepsis is characterized by a host response to a pathogen with or without organ dysfunction and is diagnosed clinically, although the exact definition and criteria for diagnosis remain under intense debate (Singer et al., 2016). Sepsis may progress into septic shock, which carries a high mortality due to poor perfusion of organs and subsequent organ failure (Rhee et al., 2019). Several mechanisms contribute to the development and progression of ALI/ARDS, including vascular injury, widespread immune cell activation, cytokine release, and thrombosis (Matthay et al., 2019). These mechanisms may synergistically contribute to vascular injury as inflammation and pro-inflammatory cytokines damage endothelial cells (Sprague and Khalil, 2009). Trauma to endothelial cells directly from pathogens or cytokines may in turn contribute to platelet adhesion and aggregation (Stokes and Granger, 2012). During ALI/ARDS, the lungs are often affected due to high vascularity and in the case of respiratory infections, proximity to the pathogenic source. Subsequent damage to the lung vasculature increases permeability to plasma protein and fluid, interstitial/alveolar inflammation, and extravascular fibrin deposition that ultimately contribute to poor respiratory status and mortality (Sibbald et al., 1981; Meduri et al., 1995; Frantzeskaki et al., 2017).

## Contributions of Inter-Endothelial Junctions to EC Barrier Function

In the healthy state, pulmonary capillaries deliver deoxygenated blood to alveoli which facilitate gas exchange (Wagner, 2015). The capillary lining of alveoli consists of a single layer of endothelial cells which form a tight protective barrier that restricts passage of large molecular weight macromolecules and fluid (Lampugnani, 2012; Komarova et al., 2017). Like single-layer epithelium, endothelial cells form inter-cellular junctions that restrict diffusion of large proteins and fluid from the intravascular space (Lampugnani, 2012). Inter-endothelial junctions vary in organization and filtration properties between vascular beds, with endothelial cells in the brain and central nervous system demonstrating the most restrictive barrier function of all blood vessels (Sweeney et al., 2019). There is wide heterogeneity between endothelial cells, with a recent study suggesting that vascular endothelium exhibits tissue-specific gene expression affecting barrier properties (Jambusaria et al., 2020). Inter-cellular junctions include adherens junctions, tight junctions, and gap junctions. We will briefly review the role each type of junction in lung endothelial cells. Characteristics of endothelial junctions across vascular beds and the contributions of junctional proteins to vascular barrier function has been reviewed elsewhere (Komarova et al., 2017).

Adherens junctions are formed by vascular endothelial cadherin (VE-cadherin), catenin proteins (α, β, and p120) and plakoglobin (Lampugnani et al., 1995; Duong and Vestweber, 2020). The junctions are anchored to the cell cytoplasm via actin filaments and intermediate filaments (Lampugnani, 2010). The cadherins and catenins organize into a zipper-like arrangement that promotes adhesion between cells (Reglero-Real et al., 2016; Lampugnani et al., 2018). VE-cadherin is an 90–140 kDa (variable due to glycosylation) transmembrane protein with extracellular cadherin domains at the N-terminus that mediate homophilic interactions between cells (Vincent et al., 2004; Brasch et al., 2011). Tension between endothelial cells is regulated by the presence of VE-cadherin at endothelial junctions and the rate of VE-cadherin internalization (Juettner et al., 2019). Loss of VE-cadherin prevents organization of endothelial cells into monolayers and vessel-like structures, resulting in fetal death in affected mice (Vittet et al., 1997). Moreover, mice expressing VE-cadherin mutants more prone to endocytosis exhibit microvascular hemorrhaging and have reduced survival rates (Grimsley-Myers et al., 2020). On the other hand, mutant VE-cadherin resistant to internalization protects against increased permeability in response to vascular endothelial growth factor (Broermann et al., 2011). VE-cadherin forms a complex with plakoglobin, β-catenin, and actin binding protein α-catenin (Lampugnani et al., 1995). α-Catenin might play a dynamic role in junctional integrity and actin polymerization, as α-catenin binds either beta catenin or actin but not both simultaneously (Drees et al., 2005; Yamada et al., 2005). Notably, α-catenin binding to actin prevents actin polymerization (Drees et al., 2005). Loss of β-catenin increases permeability in cultured lung endothelial cells (Sawant et al., 2011). Disruption of junctions may also occur downstream of mechanical sensor piezo1 in mice experiencing increased hydrostatic pressure and acute heart failure (Friedrich et al., 2019). Local cytoskeletal dynamics regulate paracellular permeability, as actin polymerization stabilizes cortical actin and promotes cell-cell adhesion (Prasain and Stevens, 2009). Rho GTPases RhoA and Rac1, which regulate actin stress fiber assembly, regulate endothelial junctions in an opposing manner: RhoA inhibition reduces endothelial permeability, while Rac1 inhibition increases permeability (Wojciak-Stothard et al., 2001; Timmerman et al., 2015). Neuronal (N) cadherin is expressed in endothelial cells, localizes to endothelial junctions, and regulates expression of both VE-cadherin and p120 catenin (Luo and Radice, 2005). N-cadherin recruits guanine nucleotide exchange factor Trio which promotes Rac1 mediated VE-cadherin trafficking to adherens junctions (Timmerman et al., 2015; Kruse et al., 2019).

Tight junctions are formed by transmembrane proteins [claudins, junctional adhesion molecules (JAMs), occludins], intracellular scaffolding proteins (zona occludens, occludins), and cytoskeleton binding proteins (e.g., cingulin and myosins) (Bazzoni et al., 2000; Zihni et al., 2016). Tight junction proteins contribute substantially to the blood-brain barrier but are present to a lesser extent in lung endothelial cells. Claudin-5 is nearly ubiquitously expressed across the vasculature (Morita et al., 1999). Overexpression of claudin-5 improves vascular barrier function in rat pulmonary endothelial cells (Soma et al., 2004). Similar to claudin-5, JAM-A is ubiquitously expressed across the vasculature. Loss of JAM-A increases endothelial permeability in lung endothelial cells by downregulating claudin-5 expression and inactivating Ras-related protein 1 (Rap-1) (Kakogiannos et al., 2020). Mechanistically, loss of JAM-A reduces expression of C/EBP-α which binds to the claudin-5 promoter to induce gene expression. JAM-A expression is also required for cAMP-mediated increases in claudin-5 expression in endothelial cells (Ishizaki et al., 2003; Kakogiannos et al., 2020). There is evidence of crosstalk between adherens and tight junctions in endothelial cells. VE-cadherin controls expression of claudin-5 through Akt-mediated phosphorylation of forkhead box factor FOXO1, which inhibits interaction of FOXO1 with β-catenin and restricts FOXO1-mediated transcriptional repression of the *CLDN5* gene (Taddei et al., 2008).

Gap junctions are comprised of connexin molecules. Connexins are tetraspanin integral membrane proteins that form hexamers and then migrate to the basolateral surface (Skerrett and Williams, 2017). Hexamers in adjacent cells form channels that allow transport of small molecules, including ions, nucleotides, amino acids, and other molecules (Figueroa et al., 2004). Electron microscopy studies of cells with gap junctions demonstrate close apposition of hexamers, with nearly 2–3 nm separating plasma membranes between adjacent cells (Goodenough and Paul, 2009). Of the known connexins, endothelial cells express Cx37, Cx40, Cx43, and Cx47 (Figueroa et al., 2004). Cx40 and Cx43 expression regulate both expression of ROCK1 and phosphorylation of the 20 kDa Myosin Light Chain 1, which are both associated with increased endothelial permeability (Zhang et al., 2015; Yin et al., 2019).

## Contributions of Caveolar Transcytosis to EC Barrier Function

As discussed above, inter-cellular junctions restrict diffusion of small molecular weight proteins across the endothelial barrier. Tracer studies with dextrans of various sizes demonstrate that small molecules (radii <10 nm) are transported from the blood into lymphatics in a size-dependent fashion, with smaller proteins appearing in the lymphatics with greater efficiency. In contrast, dextrans with radii >10 nm appeared in the lymphatics with similar efficiency despite varying size. This strongly suggests that the endothelium permits selective passage of large macromolecules (notable exception includes brain and CNS endothelium), however, the route of transport was speculated to differ from that utilized by smaller molecules. Rather than passage of large molecules through static intracellular pores, electron microscopy studies of continuous endothelial cells demonstrate transport of large molecular weight molecules through intracellular plasmalemmal vesicles known as caveolae. These vesicles start as membrane-attached flask-shaped invaginations that subsequently internalize into the cells and migrate to the opposing membrane, delivering content to the outside of the cell (Milici et al., 1987; John et al., 2003). This process is defined as transcellular transport or *transcytosis*. Transcytosis is observed primarily in endothelial and epithelial cells and can occur through caveolae, clathrin-coated pits, or other mechanisms (Tuma and Hubbard, 2003).

Among other functions, endothelial cells maintain organ integrity and function by restricting extravasation of fluid and macromolecules from the vasculature into the interstitial and alveolar space (Komarova et al., 2017). Thus, endothelial cells maintain vascular barrier function, preventing widespread edema and multi-organ dysfunction. Since the 1950s, seminal studies utilizing electron microscopy have demonstrated that caveolae are the predominant mediators of macromolecule transport in healthy endothelial cells (Palade, 1953). Several ligands have been shown *in vivo* to be transported by caveolae rather than clathrin-coated pits or paracellular transport (Simionescu et al., 1973; Milici et al., 1987; Wagner and Chen, 1991). Moreover, deletion of caveolin-1 prevents internalization of specific ligands into the cell and consequently, transcellular transport of ligand (Schubert et al., 2001; Sun et al., 2011). Inhibition of exocytosis restricts deposition of ligand but not internalization, providing strong evidence that macromolecules are transported primarily via caveolar vesicles upon internalization and subsequent trafficking through endothelial cells (Predescu et al., 1994, 1997; Schnitzer et al., 1995a). Importantly, experiments performed *in vivo* strongly suggest that caveolae-mediated uptake and transport of circulating macromolecules eventually results in deposition of the internalized cargo (macromolecular ligand) into the sub-endothelial space (Vogel et al., 2001). Thus, the majority of ligand that enters the cell is trafficked into the interstitial and parenchymal tissue. Endothelial transcytosis can be organized into three main steps: endocytosis (internalization of the vesicle), vesicular trafficking, and exocytosis (fusion of vesicle with abluminal membrane and release of contents into the extracellular space).

In lung endothelial cells, caveolae are the primary mediators of transcytosis and are responsible for delivering most macromolecules across the lung vascular wall (Jones and Minshall, 2020). In this review, we will focus on the important contributions of caveolae as transport organelles and the role of transcytosis during ALI/ARDS. We will also review potential therapeutic approaches that may (1) utilize increased transcytotic activity during ALI/ARDS for drug delivery or (2) limit endothelial transcytosis by inhibiting platelet activation and subsequent ICAM1 expression on endothelial cells. These strategies may improve recovery while reducing severity and mortality of ALI/ARDS.

## Biochemical Composition of Caveolae

Lung endothelial cells contain an abundant number of 40–80 nm, omega-shaped membrane-attached vesicles typically present as a linear array at the luminal surface (Palade, 1953). These vesicles are known as caveolae and are found in a variety of cells, including endothelial, epithelial, fibroblasts, adipocytes, and smooth muscle cells (Cheng and Nichols, 2016; Parton, 2018). There is a high degree of variability in caveolae density among the different cell types, with venous endothelium expressing the most caveolae among cell types studied and reported in the literature (Simionescu et al., 1974; Simionescu and Simionescu, 1984). In addition, caveolae exhibit different functions in different cell types (Cheng and Nichols, 2016). In addition to transport, caveolae function as signaling centers that recruit receptors, kinases, glycosphingolipids, and other molecules that contribute to endothelial cell and organ homeostasis (Parton, 2018). At the luminal surface, caveolae are identifiable by characteristic intermittent ridges on the cytosolic surface of the endothelial cell plasma membrane when viewed by scanning electron microscopy (Peters et al., 1985).

Caveolae are important for cell development and maintenance of cellular functions (Parton, 2018). These vesicles are comprised of specialized constituent proteins termed “caveolins” and “cavins” due to their localization within or associated with the caveolar membrane and relevance to caveolar stability, shape, and function (Sowa, 2012). Lung endothelial cells express caveolin-1 and caveolin-2, whereas muscle cells also express caveolin-3 (Tang et al., 1996; Razani et al., 2002; Kogo et al., 2004). Caveolin-1 is a 22 kDa scaffolding protein that is required for caveola formation in non-muscle cells. Loss of caveolin-1 prevents formation of caveolae and thus eliminates transcytosis (Schubert et al., 2001). Caveolin-1 via its scaffolding domain recruits endothelial nitric oxide synthase, which binds L-arginine as a substrate and generates nitric oxide (Bernatchez et al., 2005). Caveolin-1 deletion also leads to increased production of reactive nitrogen and oxygen species, nitrosylation of endothelial junctional proteins, and increased paracellular permeability (Siddiqui et al., 2011). Interesting, caveolin-2 deletion in mice has no effect on caveolae formation but causes lung endothelial proliferation and exercise intolerance (Razani et al., 2002). Deletion of caveolin-3 results in T-tubule abnormalities (Bryant et al., 2018), whereas loss of cavin-1 and cavin-2 results in loss of lung endothelial caveolae (Liu et al., 2008; Hansen et al., 2013). Cavin-2 regulates formation of caveolae in the lungs and adipose tissue but does not alter vesicle abundance in the heart which strongly implicates cavin-2 as having tissue specific function in the biogenesis and/or formation of caveolar vesicles (Hansen et al., 2013). Cavin-3 regulates abundance of caveolae in smooth muscle, while a recent study suggests that Cavin-4 (expressed in muscle cells exclusively) regulates T-tubule maturation in developing organisms (Zhu et al., 2017; Lo et al., 2021). Thus, caveolins and cavins regulate both caveolae abundance and function in a variety of cell types. Alteration in expression of these proteins can be found in many conditions, including ALI, cancer, and ischemia (Burgermeister et al., 2008; Zhang et al., 2016; Oliveira et al., 2017).

Glycosphingolipids, cholesterol, enzymes, caveolins, cavins, and other molecules are concentrated in lipid rafts (Parton, 2018), and in presence of caveolin-1, form caveolae (Razani et al., 2001). These specialized lipid rafts facilitate signaling events that modify cellular activity, including transcytosis, migration, nitric oxide production, proliferation, and apoptosis. Loss of lipid rafts via cholesterol depletion or caveolin/cavin deletion alters caveolae formation and/or function (Dreja et al., 2002; He et al., 2016). For example, caveolin-1 deletion results in dysregulation of endothelial nitric oxide synthase, resulting in peroxynitrite formation and disturbance of endothelial junctions (Miyawaki-Shimizu et al., 2006; Siddiqui et al., 2011). Together with coat proteins, macromolecules recruited to the caveolar microdomain regulate formation, shape, internalization and trafficking of caveolae (Sverdlov et al., 2009; Shu and Shayman, 2012; Jones et al., 2020).

## Receptor Mediated Endocytosis of Membrane Caveolae

Endocytosis is thought to be mediated by receptor-ligand interactions, but “fluid-phase” (non-receptor mediated) uptake also occurs in endothelial cells. Receptor-ligand interactions may result in subsequent fluid-phase uptake, suggesting that the two mechanisms are linked (John et al., 2003). Circulating macromolecules (e.g., albumin, LDL, viruses, bacteria, and cytokines) bind their cognate receptor localized in or near caveolar vesicles (Schnitzer and Oh, 1994; Jiao et al., 2013; Park and Lee, 2013; Shang et al., 2016). In the case of albumin, ligand-receptor interactions result in clustering of the albumin receptor gp60 and signaling via G_βγ_ unit (Tiruppathi et al., 1997; Shajahan et al., 2004b). These actions result in downstream activation of eNOS and nitric oxide (NO) production (Alpern et al., 1983; Maniatis et al., 2006; Chen et al., 2012). NO subsequently drives auto-phosphorylation of non-receptor tyrosine kinase c-Src at tyrosine 416. Indeed, loss of eNOS prevents calcium-mediated Src activation and reduces transcytosis of albumin (Chen et al., 2018). Once activated, c-Src phosphorylates caveolin-1, the essential coat protein that regulates caveolar formation in endothelial cells (Li et al., 1996; Labrecque et al., 2004). Under basal conditions, caveolin-1 forms higher order oligomers that regulate the shape of the caveolar vesicle (Zimnicka et al., 2016). Phosphorylation of caveolin-1 via activated Src causes the destabilization or loosening of caveolin-1 oligomers, which in turn, enable the swelling of caveolae, ligand uptake, and ligand internalization (Zimnicka et al., 2016). Forced expression of phospho-mimetic caveolin-1 mutant Y14D increased the number of internalized caveolar vesicles, the size of caveolae, and the amount of albumin internalization in lung endothelial cells compared to WT caveolin and phospho-defective Y14F mutant expression (Zimnicka et al., 2016). Src-mediated phosphorylation of caveolin-1 depends on palmitoylation of caveolin-1 at C156, as C156S expression mutants prevent both caveolin-1 phosphorylation and ligand endocytosis (Lee et al., 2001). In addition to caveolin-1, activated Src also phosphorylates dynamin-2 at tyrosine-231 and tyrosine-597, causing dynamin-2 to self-oligomerize and increase its interaction with caveolin-1 near the neck of membrane attached caveolae (Shajahan et al., 2004a). Dynamin-2 is a GTPase which upon phosphorylation, hydrolyzes GTP to promote fission of the caveolar vesicle from the membrane (Schnitzer et al., 1996; Henley et al., 1998). Indeed, dynamin-2 is required for fission as dynamin mutants or dynamin inhibitors prevent caveolar endocytosis (Oh et al., 2012; Triacca et al., 2017; Ali et al., 2019).

**FIGURE 1:**
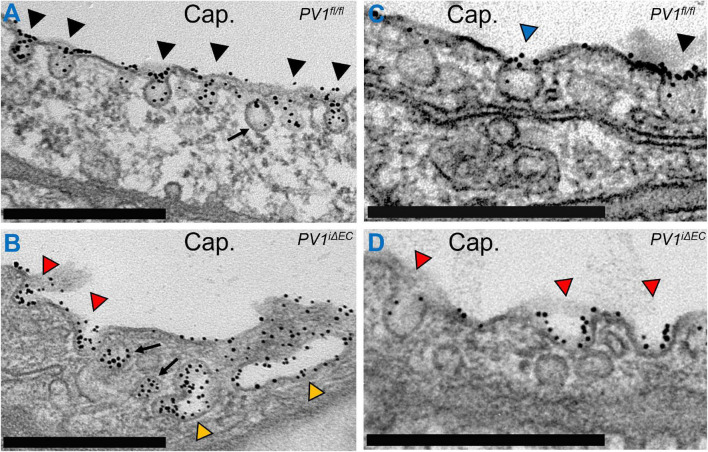
Caveolar neck protein PV1 regulates vesicle shape and internalization. Gold-labeled (Au) albumin was infused into control (*PV1^fl/fl^*) and PV1 deficient (*PV1^iΔEC^*) mice for 15 min. Lungs were subsequently harvested, minced, fixed, and further prepared for electron microscopy. **(A)** Endothelial cells contain an abundant number of caveolar vesicles (black arrowheads). Most vesicles are present on the luminal and abluminal membranes. Here, caveolae are observed filled with Au-Albumin present in the capillary lumen (Cap.). An internalized vesicle is noted (arrow) inside the cell and will ultimately migrate to the abluminal surface to deposit its content. **(B)** Loss of PV1 in endothelial cells increases vesicle internalization, noted by fewer caveolae at the cell membrane and increased number of vesicles (arrows). Loss of PV1 also increases caveolae clusters (yellow arrowheads). **(C)** A survey of lung endothelial cells reveals caveolae that feature diaphragms (blue arrowhead) comprised of PV1 oligomers and caveolae without diaphragms (black arrowhead). **(D)** Loss of PV1 results in loss of diaphragms and change in caveolar shape (red arrowheads). Observable changes include increased vesicle neck width, increased vesicle depth, and increased vesicle filling. Scale bar, 0.5 μm.

While the events described above comprise the canonical mechanism concerning receptor-mediated endocytosis, several studies suggest that caveolae recruit additional proteins to the neck region which regulate endocytosis and trafficking. Lung endothelial caveolae and caveolae in other continuous-type endothelial cell-lined vascular beds (to a lesser extent) express 5–7 nm thick diaphragms at the neck region (Stan, 2005). These diaphragms appear similar in electron micrographs to those found in fenestrated endothelial beds of abdominal organs (especially intestines and kidneys) (Stan, 2007). However, diaphragms in continuous-type endothelium appear to have a different biochemical composition when compared to those found in fenestrated endothelium (Simionescu et al., 1981; Pino, 1986). The only known protein component of the diaphragms is plasmalemmal vesicle associated protein (PLVAP, PV1), a 60 kDa protein which forms higher order oligomers that contribute to the formation and spoke-like structure of the diaphragms (Stan et al., 1999a,b). The role of PV1 in endothelial caveolae has long remained elusive but critical for human embryonic development and survival (Herrnberger et al., 2012; Stan et al., 2012). Endothelial deletion of PV1 in adult mice increased lung vascular permeability primarily of albumin by enabling an increase in its internalization (Jones et al., 2020). Moreover, loss of PV1 profoundly affected caveolar shape, increased the number of internalized caveolar vesicles, and increased caveolar cluster formation (Figure 1). Taken together, PV1 appears to restrict vascular permeability by limiting caveolar transport. Additional proteins that localize to the neck domain have also been shown to regulate transcytosis. The short isoform of intersectin-1, termed intersectin-1s, forms a scaffold at the neck region that interacts with dynamin-2 and SNAP-23 to mediate vesicle fission (Predescu et al., 2003).

## Cytoskeletal Dynamics During Vesicular Trafficking

Electron microscopy studies have demonstrated that caveolae lie near cortical actin filaments (Rohlich and Allison, 1976; Singer, 1979). Given these findings, it has long been suggested that caveolae are anchored to the cell through associations with the cellular cytoskeleton. Subsequent studies have demonstrated the regulatory role of actin filaments and microtubules in caveolar trafficking (Echarri and Del Pozo, 2015). Actin is a globular protein that polymerizes into filaments that modulate cell shape, membrane remodeling, cell contraction and cell migration (Dominguez and Holmes, 2011). In labeled cells, caveolin-1+ vesicles co-localize with cortical actin (Mundy et al., 2002). Further, actin depolymerization promotes widespread internalization of caveolin-1+ vesicles (Mundy et al., 2002). Accordingly, loss of actin polymerization following cytochalasin D increased albumin transport across lung endothelial cells *in vivo* and caused increased pulmonary edema in perfused rabbit lungs (Shasby et al., 1982). In vascular isolated smooth muscle cells, actin polymerization also increases total cellular levels of caveolin-1 and cavin-1, while depolymerization has the opposite effect (Krawczyk et al., 2015). Although characterized to a lesser extent than actin filaments, some evidence exists that microtubules maintain caveolae at the cell surface. Microtubules are formed from polymerization of tubulin proteins, resulting in larger filaments (∼25 nm) in comparison to actin filaments (∼6 nm) (Knossow et al., 2020). In contrast to actin depolymerization, loss of microtubules increases the number of membrane-bound caveolar vesicles *in vitro* (Mundy et al., 2002). Taken together, these findings indicate that cytoskeletal dynamics regulate trafficking of caveolae. A number of important questions remain regarding cytoskeletal dynamics during endocytosis and immediately following fission. Biochemical studies have shown that depolymerization of either actin or microtubules perturbs lipid raft composition and reduces caveolin co-localization with the cytoskeleton (Head et al., 2006). Caveolin-1 interacts with actin-binding proteins, particularly its phosphorylated form phospho-caveolin (Thomsen et al., 2002; Sverdlov et al., 2009). Caveolae in turn may regulate cytoskeletal dynamics, as overexpression of caveolin-1 increases microtubule polymerization, whereas down-regulation has the opposite effect (Kawabe et al., 2006).

In addition to receptors and signaling effectors, caveolae recruit proteins known to regulate actin filaments and microtubules. To date, interactions between actin proteins and caveolae have been better delineated in the literature compared to interactions between microtubules and caveolae. Rho GTPases, ∼21 kDa proteins known to regulate the cell cycle and actin polymerization, have been shown to localize to caveolae/lipid rafts (Ellis and Mellor, 2000). Rac1 forms a WAVE regulatory complex with WASP family members and promotes actin polymerization via Arp2/3 complex (Chen et al., 2017; Hinze and Boucrot, 2018). RhoA mediates actin polymerization via mDia1 and ROCK signaling (Maekawa et al., 1999; Watanabe et al., 1999). Cdc42 signals to neuronal WASP and promotes actin polymerization via Arp2/3 complex (Rohatgi et al., 2000; Hussain et al., 2001). The role of Rac1 in caveolar transcytosis has been studied in intestinal epithelial cells, where loss of Rac1 impaired the transcytosis of *Salmonella* (Lim et al., 2014). Moreover, Rac1 is required for transcytosis of horseradish peroxidase in brain endothelial cells (Coelho-Santos et al., 2016). Similarly, RhoA mediates transcytosis of *Escherichia coli* across brain endothelial cells (Khan et al., 2002; Zhang et al., 2002). The role of Cdc42 in transcytosis is less defined in the literature, however, inhibition of Cdc42 via caveolin-1 appears to promote caveolae-mediated endocytosis (Shitara et al., 2019). This corresponds to findings that Cdc42 negatively regulates endocytosis in acinar cells of salivary glands (Shitara et al., 2019). Moreover, Cdc42 acts downstream of intersectin 2L, a guanine nucleotide exchange factor, to promote actin polymerization and reduce vesicle internalization in lung endothelial cells (Klein et al., 2009). Filamin A, an actin-binding scaffold protein, is recruited to caveolar vesicles via phospho-caveolin-1 following Src activation and is required for albumin transcytosis across lung endothelial cells (Sverdlov et al., 2009). Furthermore, phosphorylation of filamin-A via PKC at serine 2152 appears to be required for internalization and trafficking of caveolae (Muriel et al., 2011). The Ras related small GTPase RalA stimulates phospholipase D mediated-phosphatidic acid production and forms a complex with caveolin-1 and filamin A in lung endothelial cells exposed to albumin (Jiang et al., 2016). Loss of RalA or inhibition of phospholipase D prevents internalization of albumin (Jiang et al., 2016).

Recently, caveolar neck proteins have been implicated in cytoskeletal remodeling and vesicle internalization. Eps15 homology domain-containing 2 (EHD2), a member of the ATP-binding dynamin family EHD proteins, has been shown to form oligomers at the caveolar neck region which restrict fission and vesicle trafficking (Stoeber et al., 2012). Loss of EHD2 or its ATP binding protein increases caveolae internalization (Moren et al., 2012; Hoernke et al., 2017). EHD2 binds Protein Kinase C and Casein Kinase Substrate in Neurons-2 (pacsin2), which complexes with F-actin (Moren et al., 2012; Kostan et al., 2014). Pacsin2 regulates vesicle shape, neck diameter and vesicle depth (Hansen et al., 2011; Senju et al., 2011). PKC phosphorylates pacsin2 resulting in reduced localization of pacsin2 to caveolae and caveolae internalization (Senju et al., 2015). In addition, EHD2 regulates eNOS function, as loss of EHD2 in endothelial cells impairs NO production (Matthaeus et al., 2019).

## Tethering, Fusion and Exocytosis of Caveolae

In endothelial cells and other polarized cells, caveolae are trafficked across the cytoplasm to the opposing membrane to which the vesicles fuse and deposit their contents outside the cell. This process is referred to as *exocytosis.* In contrast, caveolae trafficking in non-polarized cells (e.g., fibroblasts) often involves delivery of vesicles to subcellular organelles (e.g., lysosomes, proteasomes, etc.) (Parton, 2018). The precise mechanisms regarding vesicle targeting of the plasma membrane, fusion, and delivery of contents outside of the cell are not well understood. Fractionation studies have shown that endothelial cells like other vesicle-containing cells (e.g., neurons) express proteins that facilitate exocytosis, including *N*-ethylmaleimide sensitive fusion protein (NSF), soluble NSF attachment protein receptor (SNARE) proteins, and synaptosomal-associated proteins (SNAPs) (Schnitzer et al., 1995b; Predescu et al., 2001; Yamakuchi et al., 2008). Internalized vesicles contain v-SNARE proteins which interact with membrane-associated target (t) SNARE proteins and likely function to promote fusion of vesicles with the membrane (Predescu et al., 2001). As mentioned previously, SNAP-23 promotes fusion to the abluminal membrane and exocytosis of the vesicular content into the sub-endothelial space (Predescu et al., 2005). Although not well characterized in endothelial cells, 7–8 proteins (Exo70, Exo84, Sec3, Sec5, Sec6, Sec8, Sec10, and Sec15) form an exocyst complex which appear to initiate tethering of vesicles to the membrane (Wu and Guo, 2015; Martin-Urdiroz et al., 2016). One of the exocyst proteins, Sec15, interacts with Rab11 which has been shown to regulate tethering of vesicles to the membrane and exocytosis of vesicle content in endothelial cells (Wu et al., 2005; Takahashi et al., 2012). Exo70 appears to interact with Rho GTPase Cdc42 which appears to be required for exocytosis (Wu et al., 2010) family-member.

## Endothelial Transcytosis Contributes to Pathogenesis of Acute Lung Injury

Several studies have demonstrated that ALI/ARDS is associated with vascular dysfunction (Ryan et al., 2014). In response to pathogens, trauma, or widespread inflammation, the endothelium becomes hyper-permeable thereby allowing extravasation of proteins and fluid (Matthay et al., 2019). Endothelial cells may be directly injured by pathogens (e.g., *E. coli* LPS and SARS-Cov-2) or indirectly injured via widespread cytokine-mediated endothelial cell activation and injury (Cheng et al., 2017; Varga et al., 2020). Endothelial injury can progress to cell death and the extent of lung endothelial cell depletion is associated with mortality (Liu et al., 2019). Disruption of endothelial barrier function is thought to be a primary contributor to the development of ALI/ARDS and is associated with increased mortality (Komarova et al., 2017). Since endothelial barrier function is regulated by both endothelial junctions and transcytotic activity, the hyperpermeability state associated with ALI/ARDS likely involves alterations of both the paracellular and transcellular pathways. Important questions remain about the relative contributions of each pathway following lung injury. Most preclinical studies evaluate the extent of hyperpermeability rather than the route of protein and fluid transport following lung injury. Given that ALI arises from several distinct etiologies, the relative contributions of each pathway may differ widely in accordance with the variable natural history of disease progression.

Loss of endothelial junction integrity following inflammation has been well described in the literature. In cultured cells, inter-cellular junctions dissemble within minutes upon exposure to permeability factors thrombin, histamine, and VEGF-A (Rabiet et al., 1996; Ashina et al., 2015; Ourradi et al., 2017). However, in animal models of lung injury, significant vascular permeability defects are observed hours after LPS delivery or cecal ligation puncture, with the greatest differences in permeability observed between 6 and 24 h after injury (Gill et al., 2014). These findings likely reflect immediate changes to permeability in response to injury (e.g., disrupted junctions), apoptosis/pyroptosis, and transcriptional responses mediated by inflammatory signaling. Recent studies show that VE-cadherin, the adhesion protein required for formation of adherens junctions, is transcriptionally repressed by the inflammatory cytokine interleukin-1β following LPS-induced ALI in mice (Xiong et al., 2020). In contrast to adherens junctions, gap junctions may facilitate rather than prevent endothelial barrier disruption following ALI. Loss of Cx40 limits thrombin-induced endothelial disruption in cultured lung endothelial cells (Yin et al., 2019). Similarly, connexin inhibitor carbenoxolone prevented sepsis induced pulmonary vascular leakage in mice that underwent cecal ligation puncture (O’Donnell et al., 2014).

**FIGURE 2:**
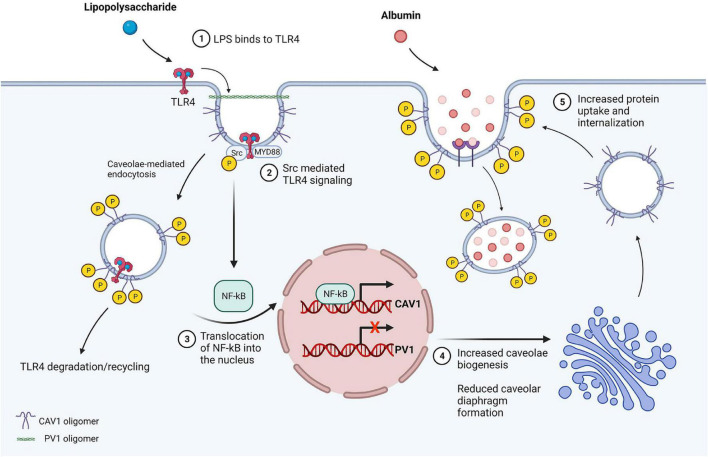
LPS-mediated inflammatory signaling increases transcytosis in endothelial cells. (1) LPS present in the lumen binds to TLR4 in caveolar microdomains. (2) TLR4 signaling activates MyD88 and Src kinase, resulting in caveolin-1 phosphorylation at tyrosine-14 and reduction in caveolin-1 oligomer stability. The vesicle is internalized via dynamin-mediated fission and TLR4 is subsequently degraded or recycled. (3) NF-κB is activated downstream of MyD88, resulting in translocation of NF-κB into the nucleus and p65/RelA-mediated upregulation of caveolin-1 expression. LPS also reduces PV1 expression, however, the mechanism through which this occurs is unclear. (4) Upregulation of caveolin-1 increases formation of vesicles, while less caveolar diaphragms are generated due to lack of PV1, which is essential for diaphragm formation. (5) Caveolae traffic to the cell membrane and invaginate, exhibiting wider neck and bulb diameter in the absence of diaphragms resulting in greater filling of the vesicle. Vesicles subsequently undergo fission, contributing to increased protein permeability of the endothelial barrier resulting in protein rich edema formation and ALI.

There is a growing number of studies highlighting the contributions of the transcellular permeability pathway upon exposure of cultured endothelial cells to inflammatory mediators and following *in vivo* lung injury. High resolution electron microscopy studies of rabbit lung endothelium revealed that there is increased abundance and internalization of caveolae immediately following intratracheal instillation of LPS (Heckel et al., 2004). In this study, 90% of tracer transport was mediated by transcytosis. This occurred 2 h after LPS delivery and was associated with thickened interstitial spaces and development of lung edema (Heckel et al., 2004; Tiruppathi et al., 2008). Similarly, *in vitro* studies have shown that LPS increases caveolin-1 phosphorylation and transcytosis prior to disruption of junctions (Wang et al., 2015). In agreement, LPS-mediated acute lung injury in mice is associated with increases in caveolin-1 phosphorylation (Oliveira et al., 2017). Loss of caveolin-1 or inhibition of caveolin-1 phosphorylation results in less albumin transport, less edema formation, and improved survival in mouse models of ALI (Garrean et al., 2006; Ivanciu et al., 2006; Jiao et al., 2013). Toll-like receptor 4 (TLR4), a pattern recognition receptor found in endothelial cells, is recruited into caveolar microdomains after binding LPS (Triantafilou and Triantafilou, 2002; Triantafilou et al., 2004). Loss/mutation of either TLR4 or caveolae (via caveolin-1 deletion) results in less injury and improved survival in models of ALI (Qureshi et al., 1999; Jiao et al., 2013). LPS induces TLR4-mediated activation of MyD88 and downstream activation of nuclear factor kappa B, resulting in increased caveolin-1 expression (Figure 2). PV1, which was recently shown to regulate caveolar uptake and internalization, is down-regulated acutely (<6 h) following LPS-mediated lung injury in mice, which may contribute to the immediate increase in caveolar transport following ALI (Jambusaria et al., 2020; Jones et al., 2020). Moreover, inhibition of exocytosis via competitive inhibition of SNAP-23 prevents ALI (Bai et al., 2015). These studies indicate that endothelial cells upregulate caveolae-mediated transcytosis in response to endotoxin and/or acute lung injury. In turn, an increase in transcytosis likely contributes to the overall loss of protein from the intravascular space and accumulation in the lung parenchyma, further driving fluid leakage via the Starling effect (Levick, 2004). Inflammation likely indirectly increases endothelial transcytosis, as neutrophil binding to intercellular adhesion molecule 1 (ICAM1) *in vivo* increases endothelial permeability to albumin (Hu et al., 2008). Similarly, hydrogen peroxide, which is produced from superoxide released from activated neutrophils, increases albumin transcytosis in mice by promoting caveolin-1 phosphorylation (Sun et al., 2009).

Procoagulant and platelet activation factors may further promote endothelial transcytosis. Notably, several of these factors have been found in the plasma of patients with ALI/ARDS. Indeed, thrombin, via caveolin-1-regulated activation of protease-activated-receptor-1 (PAR1) has been shown to stimulate albumin transcytosis via increased acid sphingomyelinase (ASM) activity and ceramide production (Kuebler et al., 2016). In the coagulation pathway, thrombin converts fibrinogen to fibrin and promotes the cross-linking of fibrin to generate clots (Mann et al., 2003). Endotoxin stimulates platelet aggregation and inflammation activates the coagulation pathway, resulting in clot formation intravascularly and in the parenchyma as evidenced by fibrin deposition in lung alveoli (Zhang et al., 2009; Foley and Conway, 2016). In addition, platelet activating factor (PAF) also increases lung vascular permeability via activation of ASM and ceramide production (Burhop et al., 1986; Uhlig et al., 2005; Yang et al., 2010). The PAF receptor (PAFR) localizes to caveolae, and disruption of lipid rafts prevents PAFR-mediated signaling through the ERK/MAPK pathway (Poisson et al., 2009). Ceramide production in endothelial cells increases exocytosis of Weibel-Palade bodies and von Willebrand factor (vWF) secretion (Bhatia et al., 2004). Elevated levels of vWF, which facilitates platelet adhesion, have been reported in patients with ALI and is associated with increased mortality (Ware et al., 2004). High mobility group box 1 (HMGB1), a non-histone chromosomal protein, is released by macrophages during sepsis where it is thought with an emerging role in the development of thrombosis, to act as a pro-inflammatory cytokine (Vogel et al., 2015; Karakike et al., 2019). HMGB1 increases albumin transcytosis in lung endothelial cells *in vitro* by binding to the receptor for advanced glycation end products (RAGE) resulting in Src activation followed by caveolin-1 phosphorylation (Shang et al., 2016). Taken together, these data suggest the possibility that increased ceramide production in the setting of endothelial inflammation/injury drives transcytosis and SNAP-mediated exocytosis of vWF resulting in increased endothelial permeability and platelet aggregation.

The recent emergence of SARS-Cov-2, a novel coronavirus belonging to the beta-coronavirus genus, has resulted in an urgent need to understand the underlying mechanisms concerning viral entry, replication, and apoptosis/necrosis of affected cells (Hu et al., 2021). The clinical syndrome associated with SARS-Cov-2, termed COVID-19 by the World Health Organization, ranges in severity from mildly symptomatic (e.g., cough and body aches) to ALI/ARDS, multi-organ system failure, and death (Goyal et al., 2020; Guan et al., 2020). The available evidence suggests that SARS-Cov-2 infects endothelial cells, causing endotheliopathy, thrombosis, and cellular death (Goshua et al., 2020; Varga et al., 2020; Liu et al., 2021). Spike proteins located on the virion surface of SARS-Cov-2 interact with ACE2 which is localized in lipid rafts (Lu et al., 2008; Zamorano Cuervo and Grandvaux, 2020). Indeed, loss of membrane cholesterol inhibits SARS-Cov-2 entry (Sanders et al., 2021). There are conflicting data regarding the expression levels of ACE2 in endothelial cells, however, there is likely heterogeneity in expression between vascular beds (Lei et al., 2021; McCracken et al., 2021). In epithelial cells, SARS-Cov-2 infection activates ASM leading to increased ceramide production (Carpinteiro et al., 2020, 2021). This finding raises the possibility of a similar mechanism occurring in endothelial cells, which would presumably lead to an increase in caveolae-mediated macromolecular transport via transcytosis. Moreover, the nucleocapsid protein of SARS-COV-2 has been shown to stimulate expression of ICAM1 in endothelial cells, which suggests the possibility that SARS-Cov-2 may indirectly stimulate transcytosis by promoting neutrophil-ICAM-1 interactions (Qian et al., 2021). The widespread endotheliopathy, thrombosis and subsequent organ damage may therefore be due to in part to virus-mediated endothelial inflammation and increase in the levels of circulating pro-thrombotic proteins that drive transcytosis and platelet aggregation via vWF secretion. On the other hand, SARS-Cov-2 appears to also directly injure endothelial cells which may in turn increase vascular permeability and thrombosis. Additional studies are needed to assess the connection between transcytosis, vWF secretion, and platelet aggregation in response to SARS-Cov-2 infection.

Endothelial damage contributes to the development of ALI/ARDS secondary to multiple etiologies, including pathogens, mechanical trauma, and complications of blood product transfusions (Matthay et al., 2019). Regarding pathogens, infection and the initial inflammatory endothelial response likely vary depending on the specific pathogen, availability and quantity of cognate receptors and signaling effectors, host endothelial cell responses, and immune-modifying agents. The literature suggests that some pathogens present either in the blood or at the abluminal surface can be internalized via endocytic vacuoles and subsequently spread to different tissues. Group B *streptococcus*, a leading cause of neonatal meningitis and sepsis, enters endothelial cells via membrane-bound vacuoles (Nizet et al., 1997). Similarly, *E. coli* K1 and *Citrobacter* spp., which also cause neonatal meningitis and sepsis, are transported through endothelial cells *in vitro* without altering inter-endothelial junctions (Stins et al., 2001). *Leptospira interrogans*, which causes renal, liver, respiratory, and neurological symptoms in humans, utilizes caveolin-1 dependent internalization for transport across endothelial cells *in vitro* (Li et al., 2019). Loss of caveolin-1 decreases Influenza A H1N1 internalization in cultured non-endothelial cells (Sun et al., 2010). Several additional pathogens require caveolin-1 or caveolin-2 expression and/or interaction with lipid rafts in order to infect cells (Zaas et al., 2009). Important questions remain concerning whether bacteria or viruses actually utilize caveolae to infect endothelial cells *in vivo*. Caveolae appear to be dispensable for development of ALI due to sepsis or endotoxemia, however, ALI in this setting may be in part attributable to increased activation of the immune system and subsequent cytokine release, particularly from macrophages (Feng et al., 2010; Oliveira et al., 2017). It is important to note that pathogens utilize various means, including non-caveolae cell entry, to infect endothelial cells. Once infected, activated endothelial cells may subsequently upregulate transcytosis of serum macromolecules. Dengue virus infection of endothelial cells increases caveolar transport of albumin *in vitro* (Chanthick et al., 2016, 2018). Endotoxin exposure likely increases transcytosis acutely due to binding of ICAM1 by leukocytes, and in the later sub-acute phase of ALI, by increasing caveolin-1 expression and reducing PV1 expression (Figure 2).

## Targeting Transcytosis for Drug Delivery in Acute Lung Injury

In survivors, resolution of ALI/ARDS is associated with widespread fibrosis leading to long-term impairment of respiratory function. There is a need for novel interventions that improve mortality and morbidity in patients experiencing lung injury. Due to the abundance of caveolae in lung endothelium, targeting caveolar transport may be an especially useful approach for delivering therapeutics into lung tissue. The established method for targeting lung endothelial transcytosis involves identification of cell surface proteins enriched in caveolar microdomains, developing or utilizing antibodies against these proteins, and assessing subsequent uptake across different tissues. Delivery of Aminopeptidase P (APP) antibodies resulted in lung specific uptake as APP is highly enriched in lung endothelial caveolae (McIntosh et al., 2002; Oh et al., 2007). The available literature suggests that there is heterogeneity in caveolar abundance, spatiotemporal organization, and function between tissues (Hansen et al., 2013; Raheel et al., 2019). Given the known heterogeneity between vascular endothelial beds, there is great potential for selective targeting of healthy and injured/altered endothelium especially where caveolae are abundant (Aird, 2012). Comparisons between healthy and tumor endothelium have shown that tumor-associated endothelial caveolae selectively recruit Annexin A1, thereby allowing for targeted uptake of conjugated antibodies into tumors but not normal parenchymal tissue (Oh et al., 2014). Although selective changes in caveolar protein expression following ALI/ARDS have been published (Oliveira et al., 2017), to our knowledge a detailed proteomic analysis of lung endothelial caveolae following inflammation/injury has not been reported.

As PV1 is a caveolar-associated protein expressed primarily in endothelial cells and has a relatively accessible extracellular domain, PV1 has emerged as an attractive target for investigation of drug delivery via transcytosis. Delivery of superoxide dismutase conjugated to PV1 antibodies resulted in lung uptake, reduced LPS-induced VCAM expression, and a reduction in LPS-induced cytokine secretion (Shuvaev et al., 2018). In a mouse model of pulmonary fibrosis, prostaglandin E2 conjugated to PV1 antibody reduced bleomycin-induced collagen deposition and lung expression of profibrotic proteins (Marchetti et al., 2019). Lipid nanoparticles conjugated to PV1 antibody reduced uptake of nanoparticles into the spleen and improved delivery to lung tissue (Li et al., 2020a). Further, the binding affinity of PV1 antibodies as well as the size of antibodies may alter uptake properties and tissue selectivity *in vivo* (Li et al., 2020b).

## Potential Role of Antiplatelet Therapy in Vascular Barrier Protection

Endothelial activation, injury, and cell death during ALI/ARDS is mediated by endotoxin, inflammatory cytokines, and often circulating blood cells derived from the bone marrow (Matthay et al., 2019). During ALI/ARDS, platelets (the anucleate non-red blood cells differentiated from megakaryocytes) become activated and secrete pro-inflammatory cytokines (e.g., IL-1β) which in turn increase endothelial permeability (Middleton et al., 2016, 2018). The role of platelets in ALI/ARDS is complex, as thrombocytopenia (characterized by clinically significant loss of circulating platelet number) is also associated with increased vascular permeability (Middleton et al., 2018). Thrombin-activated platelets stimulate expression of ICAM1 on the surface of endothelial cells (Gawaz et al., 2000). In patients with myocardial infarction, activated platelets release miR-320b, which is taken up by endothelial cells and can regulate expression of ICAM1 (Gidlof et al., 2013). Recently, elevated serum levels of miR-320b have been reported in COVID-19 patients, especially deceased patients when compared to survivors (Giuliani et al., 2022). As ICAM1 binding increases lung endothelial transcytosis, these findings raise interesting questions regarding the role of platelets in the development of hyperpermeability during the early phase of lung injury.

Clinical trial data raise the possibility that antiplatelet therapy can reduce vascular permeability in the setting of sepsis and ALI/ARDS. The Platelet Inhibition and Patient Outcomes trial found that in patients with acute coronary syndromes, treatment with ticagrelor reduced both recurrent ischemic events and overall mortality when compared to treatment with clopidogrel (James et al., 2009; Wallentin et al., 2009). A separate analysis of the trial data found that the improvement in mortality benefit was at least partially due to fewer deaths attributable to sepsis while on the medication (Storey et al., 2014). Ticagrelor but not clopidogrel reduced plasma TNFα levels and prevented the increase in lung permeability to fluid in mice (Sexton et al., 2018). Synthetic PAF antagonists lexipafant and TCV-309 have been tested in clinical trials but have not proven effective in reducing mortality in patients with sepsis or septic shock (Poeze et al., 2000; Suputtamongkol et al., 2000). Although there was no effect on overall mortality, TCV-309 decreased the extent of multi-organ failure in patients with septic shock and notably reduced vasopressor use and ventilation requirements (Poeze et al., 2000). Another recent study demonstrated that peptide-based targeting of leukocyte and platelet G_α13_-mediated integrin “outside-in” signaling improved survival, lung albumin uptake, and thrombosis in mice (Cheng et al., 2021). These findings occurred in the absence of bleeding or hemorrhagic events, a common complication of antiplatelet therapies. Finally, studies from our laboratory have demonstrated that direct targeting of αSNAP protein via novel G_α12_ N-terminal derived peptide inhibits the interaction between G_α12_ and αSNAP and thereby reduces vWF secretion and microvascular thrombosis in mice (Rusu et al., 2014; Bae et al., 2019). Additional research may lead to novel antiplatelet therapies that target both thrombosis and lung vascular permeability in sepsis and ALI/ARDS.

## Conclusion

ALI/ARDS is characterized by widespread loss of endothelial barrier function, interstitial edema, alveolar inflammation, hypoxemia, and impairment of respiratory function. Despite advances in supportive therapy, there remains no specific treatment to address the underlying mechanisms that contribute to the development and progression of ALI/ARDS. Lung endothelial transcytosis is mediated primarily through caveolae which transport macromolecules between the vasculature and subendothelial space. Caveolae-mediated endocytosis and subsequent transcytosis is regulated by both the phosphorylation and interaction of caveolar coat proteins and proteins located at the neck region. PV1, EHD2, and intersectin-1s specifically regulate caveolar shape and the rate of vesicle internalization in endothelial cells. Preclinical studies suggest that ALI is associated with increased transcytosis, which may occur upon direct activation of endothelial cells by infectious agents/injury or by stimulation with pro-thrombotic proteins. Targeting proteins enriched in caveolar microdomains may significantly improve transcytosis of therapeutics and thus enhance drug delivery into extravascular tissues and organs such as the lung. Patients with ALI/ARDS may benefit from lung tissue-specific therapies that limit interstitial/alveolar inflammation, edema formation, and cell death. Currently available and emerging antiplatelet therapies may reduce lung vascular permeability in the setting of sepsis and ALI/ARDS. As death from pulmonary complications comprises a significant portion of the mortality burden due to ALI/ARDS and specifically COVID-19, a greater understanding of the underlying mechanisms of lung endothelial transcytosis may result in novel treatment strategies that improve morbidity and mortality.

## Author Contributions

JJ wrote the article and generated the figures. RM provided initial direction and references that guided the thematic area and edited the article and figures. Both authors contributed to the article and approved the submitted version.

## Conflict of Interest

The authors declare that the research was conducted in the absence of any commercial or financial relationships that could be construed as a potential conflict of interest.

## Publisher’s Note

All claims expressed in this article are solely those of the authors and do not necessarily represent those of their affiliated organizations, or those of the publisher, the editors and the reviewers. Any product that may be evaluated in this article, or claim that may be made by its manufacturer, is not guaranteed or endorsed by the publisher.
